# Effects of Employee–Artificial Intelligence (AI) Collaboration on Counterproductive Work Behaviors (CWBs): Leader Emotional Support as a Moderator

**DOI:** 10.3390/bs15050696

**Published:** 2025-05-17

**Authors:** Qingqi Meng, Tung-Ju Wu, Wenyan Duan, Shijia Li

**Affiliations:** 1 Business School, Harbin Institute of Technology, Harbin 150001, China; 15045067888@163.com; 2 School of Management, Harbin Institute of Technology, Harbin 150001, China; tjwu@hit.edu.cn (T.-J.W.); 22s010100@stu.hit.edu.cn (S.L.)

**Keywords:** employee–AI collaboration, counterproductive work behavior, loneliness, emotional fatigue, leader emotional support, conservation of resources theory

## Abstract

The accelerated advancement of artificial intelligence (AI) has positioned it as a novel colleague. However, as employees collaborate with AI colleagues in daily work, their communication and interaction with human colleagues may decrease. This may result in feelings of loneliness and a potential reduction in emotional resources, potentially leading to counterproductive work behavior (CWB). Drawing from the conservation of resources (COR) theory, we hypothesize that employee–AI collaboration may amplify employees’ CWB due to loneliness and emotional fatigue. The potential mitigating effects of leader emotional support on these outcomes are also considered. To test these hypotheses, a 2 × 2 vignette experiment (N = 167) was conducted. The results demonstrate that employee–AI collaboration exerts a substantial positive influence on loneliness. Loneliness further increases employees’ emotional fatigue, which in turn increases CWB. Leader emotional support—the care and motivation demonstrated by leaders has been identified as a key factor in reducing loneliness. This research contributes to the extant literature on employee–AI collaboration and CWB, and expands the application scope of COR. Practical implications arise for managers, who are encouraged to consider the impact of employee–AI collaboration on interpersonal interaction and to address employees’ emotional needs in a timely manner.

## 1. Introduction

Organizations across the globe are experiencing significant technological shifts brought about by the advent of artificial intelligence (AI), a phenomenon commonly referred to as the Fourth Industrial Revolution. AI is distinct from software code, which is defined as a computer program that can only execute specified “if then” rule instructions (Webb, 2019). Compared to software code that guides computers or other electronic devices to perform specific routine tasks, AI is a set of flexible, adaptive, and human-like (or beyond human capabilities) interrelated technologies that can autonomously participate in learning, problem-solving, and executing target tasks (Bankins et al., 2024; Huo et al., 2025; Knani et al., 2022). The flexibility and rapid development of AI enable it to contribute to achieving enterprise intelligence, real-time, horizontal and vertical digital integration, and autonomous coordination of work processes (Kinkel et al., 2022; Mogaji et al., 2024). The integration of AI has led to the development of highly flexible and efficient working conditions, resulting in a growing trend of companies incorporating AI into their operations and promoting employee–AI collaboration—a work behavior or process in which employees engage in collaboration with AI (Kong et al., 2023; Yin et al., 2024).

The collaboration with AI has precipitated profound changes in the workplace, exerting a multifaceted influence on employee behavior and the nature of work experience (Li et al., 2024). However, extant research has primarily explored the relationship between AI and employee turnover intention, career development, knowledge hiding, satisfaction, job reshaping, etc. (Budhwar et al., 2022; Kong et al., 2023; Wu et al., 2024), and few studies have focused on the counterproductive work behavior (CWB) caused by AI (Zhou et al., 2025). CWB is defined as a range of voluntary behaviors exhibited by employees that are harmful to the organization and its members (Doan & Nguyen, 2025; Zhou et al., 2025). These behaviors are regarded as counterproductive due to their deleterious impact on organizational effectiveness, employee well-being, and overall workplace functioning (Bowling & Gruys, 2010). Organizations and managers must prioritize minimizing the generation of CWB. Therefore, an investigation was conducted into the relationship and mechanism of action between the two.

As AI becomes increasingly integrated into employee work practices and task processes, it will significantly alter the structure, nature, and meaning of the workforce, the relationship between employees and technology (Chowdhury et al., 2023; Einola & Khoreva, 2023). The collaboration with AI has led to a shift in the workplace, whereby employees are no longer the sole actors. AI has emerged as a new member of the organization, assuming the role of a partner to employees (Kong et al., 2023). Both employees and AI are engaged in different tasks and are interdependent on the same or closely related tasks, thereby achieving employee–AI collaboration (Chowdhury et al., 2023; Einola & Khoreva, 2023; Kong et al., 2023). AI collaboration has not only resulted in alterations to the manner in which tasks are completed and overall efficiency, but also has a significant effect on the emotional experiences and social needs of employees (Marriott & Pitardi, 2024; Salas-Pilco, 2020). As a social species, humans require the establishment of meaningful connections with others in their professional and personal lives to ensure the fulfillment of their emotional needs (Sullivan & Bendell, 2023). When employees fail to establish social connections with others within the organization to maintain resources, negative emotions such as loneliness can arise (Ernst et al., 2022; Luhmann et al., 2023; Sullivan & Bendell, 2023). Loneliness is a subjective psychological state experienced by individuals due to unsatisfactory interpersonal communication (Buecker et al., 2021; Hu et al., 2023; Rippé et al., 2022).

In traditional work environments, employees have the opportunity to fulfill their daily social needs and obtain emotional resources through communication and interaction with their human colleagues (Charoensukmongkol & Phungsoonthorn, 2020; Halbesleben & Wheeler, 2012). However, AI is inherently incapable of replicating the features and elements necessary for human social interaction, thereby impeding the provision of interaction and emotional feedback to employees (Dietvorst et al., 2018; Tang et al., 2023). Employees perceive a reduction in communication with others, which may subsequently lead to feelings of loneliness (Tang et al., 2023). While Tang et al. (2023) have examined the potential harm that AI use may inflict on employees’ interpersonal communication, their focus is primarily on the increase in social behavior after work as a result of reduced workplace interaction. However, given the heightened levels of loneliness that employees experience due to the decline in social interaction within the workplace, it is more plausible that these sentiments would lead to actions that directly impact their professional lives, rather than merely seeking emotional support through post-work social interactions. Consequently, this research aims to validate the mediating effect of emotions between employee–AI collaboration and CWB.

Drawing from the conservation of resources theory (COR), when employees’ resources are at a low level, they may take measures to avoid further depletion of resources or seek alternative means of supplementing them (Dong & Geng, 2023). On the one hand, employees may become passive and even late or leave early due to insufficient resources to avoid further depletion of emotional resources. On the other hand, employees may even engage in behavior that harms the interests of the organization, such as misappropriation of organizational assets, fraudulent reimbursement, or exploitation of authority to procure external resources, thereby enhancing their own resource base. In conclusion, insufficient resources for employees may result in the manifestation of various forms of CWB.

According to the COR theory, employees actively seek to supplement their resources in order to maintain an appropriate level of resources (Hollebeek et al., 2023). For instance, employees might actively seek additional interpersonal support to mitigate feelings of isolation. Leaders assume a pivotal role in this context by providing emotional resource support, which directly aids employees in restoring their emotional reserves. Also, to mitigate the adverse effects of AI collaboration on employee emotional well-being and CWB, it is imperative for leaders to fulfill their managerial responsibilities in a timely and effective manner. Therefore, the leader emotional support is a key factor in reducing the negative effects of AI collaboration on employee loneliness. By providing care and motivation, leaders can assist employees in overcoming feelings of loneliness, reducing emotional fatigue, and increasing their job satisfaction and engagement (Kock et al., 2018). To validate the theoretical model, this research designed a 2 × 2 vignette experiment, in which the levels of employee–AI collaboration and leader emotional support were manipulated. The results confirmed the chain intermediary role of loneliness and emotional fatigue between employee–AI collaboration and CWB. Additionally, the important regulatory role of leader emotional support in timely supplementing the emotional resources needed by employees in their work was confirmed.

This research has yielded both theoretical and practical contributions. Theoretically, this research integrated the COR theory with the employee–AI collaboration, thereby validating the applicability of the COR theory in elucidating employee emotional and behavioral alterations. Concurrently, this research examined the effect of employee–AI collaboration on CWB, revealing the potential negative effects of AI collaboration on employees’ work behavior and supplementing relevant research on the antecedents of CWB. Next, this research examined the impact pathways of employee–AI collaboration on CWB from an emotional perspective, thereby expanding the application scope of COR. This provides a new perspective for predicting and understanding the CWB that employees may experience when collaborating with AI. Third, this research validated the moderating effect of leader emotional support on the relationship between employee–AI collaboration and loneliness, further enriching the theoretical boundaries of COR theory. Practically, it is recommended that organizations prioritize the impact of employee–AI collaboration on employee CWB and take timely measures to mitigate CWB, in order to avoid the negative impact of AI collaboration on the organization. Second, organizations should prioritize the emotional well-being of their employees, recognizing the importance of providing them with the emotional resources necessary for effective AI collaboration. By implementing these measures, organizations can foster a conducive environment for their employees to maintain sufficient emotional resources. This, in turn, can prevent the exacerbation of emotional resource depletion and its deleterious impact on employee behavior. Finally, leaders can provide emotional support to employees, such as actively offering emotional care and interacting more with them to alleviate loneliness and emotional fatigue caused by AI collaboration.

## 2. Theoretical Framework and Hypotheses

### 2.1. Employee–AI Collaboration and CWB

The employee–AI collaboration emphasizes the process of human and AI working together to achieve collaborative development (Hai et al., 2025; Marvi et al., 2025; Yin et al., 2024). The primary objective is to ensure the security, continuity, and efficacy of the collaboration between employees and AI technology (Chowdhury et al., 2022; Kong et al., 2023; Yin et al., 2024). In the context of employee–AI collaboration, AI serves as an effective means to enhance employee productivity and work efficiency, thereby providing a competitive advantage for organizations (Chowdhury et al., 2022). However, organizations and managers must consider not only employees’ work performance but also their emotional experiences, which are critical antecedents of job performance (Duan et al., 2024). In this scenario, the integration of AI collaboration enables employees to engage with AI to a greater extent than with human colleagues during their work. This enables the automation of numerous tasks that were previously dependent on interaction and cooperation among employees (Dietvorst et al., 2018; Tang et al., 2023). Consequently, employees may encounter feelings of isolation, as their work becomes increasingly independent and reliant on technology rather than on interpersonal communication (Cacioppo et al., 2006; Rodriguez et al., 2020; Tang et al., 2023). According to the principles of the COR theory, individuals endeavor to acquire, protect, and enhance their personal resources while implementing measures to prevent the depletion or scarcity of these resources (Dong & Geng, 2023; Halbesleben et al., 2014; Hollebeek et al., 2023). Drawing from the COR theory, employees who experience loneliness tend to have a reduced level of resources at their disposal. On the one hand, employees must expend their own resources to cope with the adverse effects of loneliness, which ultimately results in further resource depletion. On the other hand, employees endeavor to identify strategies to replenish and sustain their emotional resources. However, the reduction in social interaction impedes their capacity to obtain emotional support from external sources, leading to a prolonged state of depleted emotional resources.

CWB is defined as a range of actions exhibited by employees in the workplace that have the potential to harm the interests of the organization and its members (Bhattacharjee & Sarkar, 2024; Choi et al., 2024). The term “CWB” is used to describe actions that have a negative effect on the organization or its members, regardless of whether they are intentional or violate organizational norms (Bhattacharjee & Sarkar, 2024; Dalal et al., 2009). CWB manifests in various forms, including absenteeism, tardiness, early departure, work alienation, embezzlement of property, intentional destruction, personal attacks, insults, physical harm, sexual harassment, and the spreading of rumors (Bowling & Gruys, 2010; Gruys & Sackett, 2003). In the context of this research, the significant impact of employee–AI collaboration on employees’ behavior and psychological state may lead to feelings of loneliness and depletion of emotional resources. According to the COR theory, when employees experience resource depletion, negative affect, and fatigue during their interactions with AI, they may engage in behaviors aimed at restoring their resources or reducing resource consumption. These behaviors may encompass CWB as a means to cope with the perceived loss or scarcity of resources.

### 2.2. The Mediating Role of Loneliness

Human beings are a social species, and individuals often need to establish meaningful connections with others not only in their personal lives, but also in their professional lives (Sullivan & Bendell, 2023). These connections are of critical importance for maintaining optimal mental health and overall well-being. The failure of employees to establish meaningful social connections with others (e.g., leaders or colleagues) within the organization can result in the emergence of negative emotions, such as loneliness (Ernst et al., 2022; Luhmann et al., 2023; Sullivan & Bendell, 2023). Specifically, loneliness is a subjective psychological state (Buecker et al., 2021; Hu et al., 2023; Rippé et al., 2022). Individuals may experience feelings of loneliness as a result of unsatisfactory interpersonal interaction. It is manifested as pain and isolation due to a lack of expected social relationships (Cacioppo et al., 2006; Rodriguez et al., 2020). It is important to note that loneliness is not solely a matter of physical solitude; rather, it is largely shaped by an individual’s perception, beliefs, and biases (Rodriguez et al., 2020).

The potential for collaboration between employees and AI in the workplace has the capacity to complement each other and achieve mutual benefit (Haesevoets et al., 2021). AI is progressively assuming the roles of an assistant, colleague, or team member for employees (Savela et al., 2021; Seeber et al., 2020). However, AI, as an interactive system or mode that facilitates employees in achieving task goals, is unable to elicit the same response as interpersonal interaction (Anthony et al., 2023; Moussawi et al., 2021). AI is deficient in the essential features of human social interaction, thereby impeding the provision of meaningful interaction and emotional feedback to employees (Dietvorst et al., 2018). Furthermore, the advent of AI collaboration has resulted in the partial automation of tasks and a concomitant reduction in social interaction among employees (Tang et al., 2023). Tasks that previously necessitated interaction and cooperation among employees have been supplanted by employee–AI collaboration (Hai et al., 2025; Marvi et al., 2025; Yin et al., 2024), leading to a decline in communication and emotional exchange between employees and colleagues. In summary, from the perspective of AI itself lacking emotional intelligence, or from the perspective of AI substituting for certain employees, the integration of AI collaboration will curtail employees’ interpersonal emotional communication and impede their acquisition and maintenance of emotional resources (Dietvorst et al., 2018; Tang et al., 2023). Therefore, although employee–AI collaboration enhances employees’ work efficiency and future career development (Sowa et al., 2021; Kong et al., 2023), it has nevertheless intensified feelings of loneliness and social isolation among employees, with a detrimental impact on interpersonal communication. So, we hypothesize the following:

**Hypothesis** **1.** *Employee–AI collaboration is positively related to loneliness.*

### 2.3. The Chain Intermediary Role of Loneliness and Emotional Fatigue

Under the influence of AI collaboration, loneliness, as a negative emotion, can exacerbate resource depletion for employees without emotional resource relief. Emotional fatigue is defined as a state of feeling overwhelmed and exhausted based on externally imposed emotional needs (Anand & Mishra, 2021). The COR theory posits that individuals strive to preserve the resources they deem important, and the absence or diminution of these resources can trigger stress and adverse emotional responses (Chen et al., 2021; F. Liu et al., 2019). On the one hand, the collaboration with AI in the workplace has the potential to induce long-term loneliness, which may, in turn, contribute to emotional problems such as depression, boredom, anxiety, and confusion among employees. These negative emotions continue to deplete employees’ emotional resources, which ultimately results in emotional fatigue (Anand & Mishra, 2021). In other words, employees experiencing feelings of loneliness may persist in exhibiting low emotional levels due to their incapacity to cultivate conducive social relationships within the professional milieu. This, in turn, engenders adverse emotional consequences, such as heightened dissatisfaction, and culminates in the onset of emotional fatigue (Jaremka et al., 2013, 2014). On the other hand, employees may experience an escalation in emotional demands due to feelings of loneliness (Tang et al., 2023). The importance of emotional needs in determining emotional fatigue has been demonstrated. Employees may further deplete their own resources by seeking emotional resources (Barnes & Van Dyne, 2009; X. Liu & Yu, 2019). Accordingly, based on the COR theory, this research posits that loneliness primarily contributes to employees’ emotional fatigue by leading to the depletion of their emotional resources, accelerating resource consumption, and impeding the acquisition of new resources.

Emotional fatigue has been widely acknowledged as a pivotal indicator of occupational stress and burnout, and is linked to a multitude of adverse job outcomes (Anand & Mishra, 2021; Barnes & Van Dyne, 2009; X. Liu & Yu, 2019). CWB refers to the intentional actions by employees that have the potential to cause harm to an organization, its members, or both (Banks et al., 2012; Bolton et al., 2012; Krischer et al., 2010). When employees have low levels of emotional resources, they may engage in certain behaviors to restore resource balance or reduce further resource loss (Dong & Geng, 2023; Hollebeek et al., 2023). Employees who are emotionally exhausted may cope with the depletion of their emotional resources by reducing work engagement or adopting negative behaviors. Such behaviors include arriving late or leaving early, displaying a lack of concern for work outcomes or organizational goals, poor task execution, intentionally reducing work quality or workload, and even engaging in other forms of counterproductive conduct (Gruys & Sackett, 2003; Marcus et al., 2013). Consequently, employees may intensify CWB due to emotional fatigue as a means of expressing their discontent, thereby reducing resource depletion and potentially even acquiring resource replenishment. So, we hypothesize the following:

**Hypothesis** **2.** *Loneliness is positively related to emotional fatigue.*

**Hypothesis** **3.** *Emotional fatigue is positively related to CWB.*

In conclusion, the integration of AI into the workplace will result in a reduction of CWB. This is due to the chain intermediary role of loneliness and emotional fatigue, which act as a chain between employee–AI collaboration and CWB. Therefore, we hypothesize the following:

**Hypothesis** **4.** *Loneliness and emotional fatigue play a chain intermediary role between employee–AI collaboration and CWB.*

### 2.4. The Moderating Effect of Leader Emotional Support

The profound impact of leaders on employees cannot be ignored. When employees’ emotional resources are low, leader emotional support from becomes particularly important (Wang et al., 2023). Leader emotional support refers to the care, listening, encouragement, or sympathy that employees perceive from their leaders (Lin et al., 2022). Employees with high leader emotional support mean that they have leaders who are considerate, caring, and can share their inner feelings with them. High leader emotional support provides employees with the emotional resources they need in their work, helping them improve their ability and resources to deal with problems at work (Kock et al., 2018; Lin et al., 2022). This is a way for employees to obtain external resources in their work. On the contrary, low leader emotional support and responsiveness to subordinates’ needs not only make it difficult for subordinates to cope with daily work tasks, but also reduce the extra effort they put in beyond the formal job requirements (Kurtessis et al., 2015; Shanock & Eisenberger, 2006).

**Figure 1:**
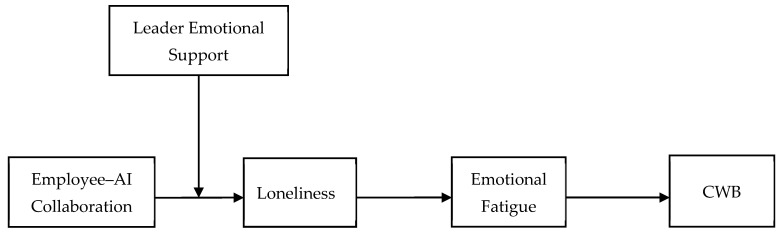
Theoretical model.

According to the COR theory, emotional support from leaders can alleviate the loneliness caused by AI collaboration by supplementing employees’ resources (Dong & Geng, 2023; Hollebeek et al., 2023). Leader emotional support can be seen as an important social resource that helps alleviate employees’ loneliness. For example, leaders create a supportive psychological environment for employees by expressing care, understanding, and encouragement. Previous research has shown that high levels of leader emotional support can provide employees with the emotional resources they need at work, thereby enhancing their ability and resources to cope with work challenges (Kurtessis et al., 2015; Shanock & Eisenberger, 2006). When leaders have high levels of emotional support, lonely employees can receive additional social support, a sense of identification, and a sense of belonging from leaders, thereby supplementing their emotional resources (Banks et al., 2012; Shanock & Eisenberger, 2006). At the same time, employees who have high leader emotional support can receive more emotional care and understanding, which helps to strengthen employees’ emotional connections to the organization and to their coworkers (Kurtessis et al., 2015; Lin et al., 2022). The emotional resources consumed in work are also restored, and the level of loneliness caused by AI collaboration is reduced (Figure 1). So, we hypothesize the following:

**Hypothesis** **5.** *Leader emotional support moderates the positive relationship between employee–AI collaboration and loneliness, such that the relationship is weaker (vs. stronger) when leader emotional support is high (vs. low).*

## 3. Method

### 3.1. Sample and Procedure

Credamo is an online research platform that is widely used for data collection (Liang et al., 2022; Wu et al., 2024). It offers functions such as questionnaire design, sample service, and statistical analysis. A total of 200 questionnaires were distributed on the Credamo platform, exploring the distinctive impact of employee–AI collaboration on employees. The research employed a 2 (high employee–AI collaboration vs. low employee–AI collaboration) × 2 (high leader emotional support vs. low leader emotional support) manipulation of employees through vignette experiments. The participants were randomly assigned to one of four experimental conditions. The vignette experience does not impose any limitations on the actual identity of the participants, nor does it require them to possess a deep understanding of the research topic prior to completing the questionnaire (Dennis et al., 2023; J.-P. Stein et al., 2020). Therefore, the participants in this study are not restricted to any specific group; they can be students, employees, or any other individuals. In each group of the vignette experiment, participants were exposed to varying levels of the manipulated factors—employee–AI collaboration and leader emotional support—while all other conditions remained constant. This methodological approach enables a comprehensive evaluation of the effects of these manipulated factors on participants while concurrently minimizing the influence of extraneous variables (Aguinis & Bradley, 2014; Cummings & Dennis, 2018; Dennis et al., 2011; Greenberg & Eskew, 1993).

The participants were initially presented with a vignette text, which they were invited to read and engage with. This text described a vignette in which the participants imagined themselves (or not) working together with AI. Subsequently, participation in this study entailed reading a paragraph about leadership, which determines whether employees could receive care and motivation from their leaders during (or in the absence of) collaboration with AI. Next, participants were required to complete the manipulation test items, thus ensuring the successful implementation of the experimental manipulation. Finally, participants were required to answer the questions about loneliness, emotional fatigue, and CWB, in addition to providing accurate responses to demographic inquiries. In accordance with the standards set forth by the American Psychological Association (APA), this study adhered to the highest standards of participant rights and protection. All participants were informed of the study’s anonymity and data confidentiality principles, which were designed to safeguard the safety and interests of participants. To encourage participation in the study, participants who complete the questionnaire will receive a reward of 3 RMB. To ensure the reliability of the data collected, we incorporated an attention check question into the survey (e.g., “For this question, please select ‘strongly agree’”) to identify and filter out inattentive responses (Wu et al., 2024). Following the exclusion of questionnaires that did not pass the attention check and had abnormal response times, such as long response times (>600 s) or short response times (<60 s), a total of 167 valid questionnaires were collected (response rate = 83.50%). Among the 167 participants, 70.70% were female (SD = 0.457). The average age of the participants was 29.976 years (SD = 6.730), and the average tenure was 6.249 years (SD = 5.631). The majority of participants (89.20%) held a bachelor’s degree or higher (SD = 0.711). The results of the descriptive statistics and correlation analysis are presented in Table 1.

### 3.2. Manipulation and Measures

*Employee–AI collaboration manipulation.* Participants were presented with a series of vignettes pertaining to the subject of employee–AI collaboration. For example, the text in the experimental group (N = 85) was “*AI is powerful and can analyze data, process results, and provide work suggestions… AI is very helpful for your work, and you will collaborate with AI to complete tasks as needed*”. In contrast, participants in the control group (N = 82) “*… AI is not very helpful for your work. It cannot help you solve the work difficulties or problems you encounter, so you hardly collaborate with AI to complete tasks*”.

*Leader emotional support manipulation.* We believe that leader emotional support may alleviate loneliness caused by employee–AI collaboration. Consequently, the level of emotional care provided by leaders towards employees was manipulated in the course of the experiment. The high leader emotional support (N = 86) text was as follows: “*... In daily work, leaders often care about your emotions and feelings, listen to the problems and difficulties you encounter in your work, and give you encouragement and support*”. Low leader emotional support (N = 81) is described as follows: “*In daily work, leaders only focus on your work results and efficiency… In the event of difficulties or problems encountered in the course of work, leaders are unlikely to offer you any form of emotional encouragement or support*”.

*CWB.* The 7-point Likert scale developed by Dalal et al. (2009) and widely used in follow-up research was used to measure the CWB (1 = strongly disagree and 7 = strongly agree). Sample item is as follows: “*I gossiped about other people at work*”. Cronbach’s α was 0.834.

*Loneliness.* The loneliness was measured using Tang et al.’s (2023) three items scale. The items were measured on a 7-point Likert scale (1 = strongly disagree, 7 = strongly agree). One of the items was “*I felt left out from one or more of my subordinates*”. Cronbach’s α was 0.890.

*Emotional fatigue.* The scale was developed by Frone and Tidwell (2015), which contains a total of 6 items; one of the sample items was as follows: “*I feel emotionally worn out at the end of the workday*”. The items were measured on a 7-point Likert scale ranging from 1 (never) to 7 (always), and Cronbach’s α was 0.934.

*Control Variables.* In light of previous research on the factors that may influence employee organizational citizenship behavior, gender, age, education, and tenure were selected as control variables (Karatepe et al., 2019).

### 3.3. Results

*Manipulation check.* The manipulation test items used the employee–AI collaboration scale developed by Kong et al. (2023). This scale was widely used to measure the collaboration between employees and AI in the workplace, as well as the degree of interaction between them (Hai et al., 2025; Marvi et al., 2025; Yin et al., 2024). At the end of the study, participants were asked “AI participates in my problem-solving process” (1 = not at all to 7 = very much). The T-test revealed that there is a significant difference (F = 30.893, *p* < 0.001) between the employee–AI collaboration group and the control group. As anticipated, the employee–AI collaboration group exhibited significantly higher evaluations of the level of collaboration (M = 5.767, SD = 0.466) in comparison to the control group (M = 2.229, SD = 1.250). Consequently, the vignette manipulation was successful.

*Mediation analyses.* The univariate analysis demonstrated that, in comparison to employees who work independently (M = 2.496, SE = 0.133), employees who collaborate with employee–AI exhibited elevated levels of loneliness (M = 2.874, SE = 0.130), F (1, 161) = 4.100, *p* < 0.05, η^2^ = 0.025, thereby Hypothesis 1 was supported. To further substantiate the influence of loneliness on emotional fatigue and its chain intermediary role, we conducted a regression analysis. Table 2 corroborates the positive effect of employee–AI collaboration on loneliness (β = 0.157, *p* < 0.05). Furthermore, loneliness has been found to lead to emotional fatigue among employees (β = 0.822, *p* < 0.001), thereby confirming Hypothesis 2. The continuous depletion of employees’ emotional resources and the exhaustion caused by the inability to replenish resources can result in behaviors that are detrimental to the organization, leading to an increase in CWB (β = 0.450, *p* < 0.01). Hypothesis 3 was confirmed. According to the mediation pathway (Table 3), the mediation effect of only considering loneliness (indirect effect = −0.003, 95% CI [−0.103, 0.108], SE = 0.051) or emotional fatigue (indirect effect = −0.008, 95% CI [−0.093, 0.069], SE = 0.040) between employee–AI collaboration and CWB was not significant. Therefore, loneliness and emotional fatigue play a chain intermediary role between employee–AI collaboration and CWB (indirect effect = 0.116, 95% CI [0.004, 0.257], SE = 0.066), and Hypothesis 4 was confirmed.

**Figure 2:**
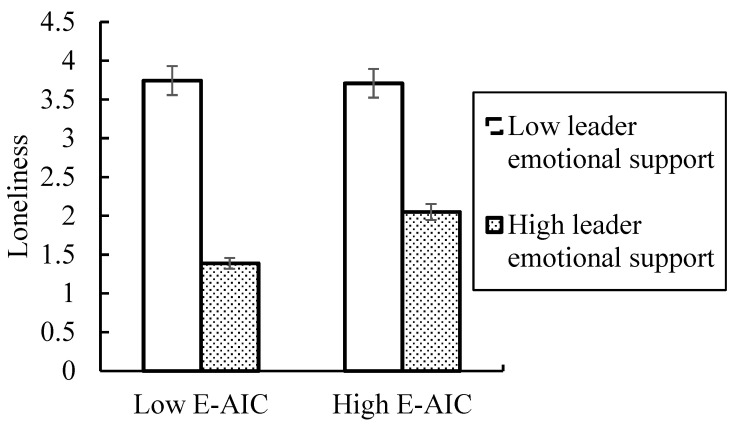
The moderating effect of leader emotional support between employee–AI collaboration (E-AIC) and loneliness.

*Moderation analyses.* A two-way ANOVA revealed a significant interaction between the employee–AI collaboration manipulation and leader emotional support manipulation predicting loneliness (F (1, 159) = 10.937, *p* < 0.01, η^2^ = 0.064), such that the effect of employee–AI collaboration on loneliness was weaker for participants who have a high level of leader emotional support (M = 2.051, SE = 0.104) than participants who have a low level of leader emotional support (M = 3.709, SE = 0.104, *p* < 0.001; Figure 2). There was a significant difference for participants in the control condition as a function of leader emotional support (high leader emotional support: M = 1.389, SE = 0.104 vs. low leader emotional support: M = 3.745, SE = 0.111; *p* < 0.001; Figure 2). Thus, Hypothesis 5 was supported.

## 4. Discussion

When it comes to the negative impacts of AI, existing research has predominantly focused on the displacement of human workers and the resulting job insecurity and anxiety (Hu et al., 2023; Suseno et al., 2022; Wu et al., 2022). However, it is important to recognize that technological advances such as AI are unlikely to completely eliminate existing occupations. Instead, they are more likely to change the way workers work, shifting the focus from traditional “human–human collaboration” to a new paradigm of “human–AI collaboration” (Fügener et al., 2022; Medici et al., 2023; Wu et al., 2022). Although Tang et al. (2023) considered the potential harm that AI use may cause to employees’ interpersonal communication, their focus is on the increased social behavior after work caused by the reduction of employees’ interpersonal interaction. Considering the increased loneliness and dissatisfaction among employees due to reduced social behavior at work, it is more likely to prompt them to do things that directly affect their work, rather than just seeking emotional support from social interactions after work. Therefore, we validated the mediating mechanism of employee–AI collaboration on CWB by influencing loneliness and emotional fatigue.

The results of the vignette experiment have confirmed that employees who engage in AI collaboration experience elevated levels of loneliness as a consequence of diminished interaction with their colleagues. As a consequence of the replacement of tasks that originally required interaction and collaboration among employees with AI collaboration, the collaboration between employees and AI reduces the communication and interaction of employees with others. Specifically, compared to human-human interaction in traditional work, employee–AI collaboration significantly reduces the frequency of interpersonal communication and interaction between employees in their work (Tang et al., 2023). However, according to Maslow’s hierarchy of needs theory, we all know that people have a need to connect and interact with others (Cui et al., 2021; Rojas et al., 2023). Therefore, the reduction of interpersonal interaction may have an impact on employees’ emotions and subsequent work behavior. This may result in feelings of isolation among employees. Furthermore, feelings of loneliness can result in a further depletion of employees’ emotional resources (Jaremka et al., 2014). Based on the COR theory, a lack of replenishment of employees’ resources will result in emotional fatigue if they are continuously depleted (Dong & Geng, 2023; Hollebeek et al., 2023; Jaremka et al., 2013). Emotional fatigue is not only associated with negative psychological states, but may also affect employee performance and behavior. Employees may engage in behaviors that are detrimental to the organization, known as CWB, in order to avoid further depletion of emotional resources. Therefore, this research validated the differences in employee emotional and behavioral performance between traditional work modes and AI collaboration modes, provides a contribution to existing research on employee–AI collaboration, and emphasizes the potential negative impact of AI collaboration on individual employees and even organizations.

Drawing upon the COR theory, when employees encounter resource constraints, they tend to adopt strategies to prevent further depletion or seek alternative means to replenish those resources (Dong & Geng, 2023; Hollebeek et al., 2023). The advent of AI collaboration invariably necessitates that employees engage in perpetual learning to acquire knowledge pertinent to this domain. In this process, the training opportunities provided by organizations for employees play a crucial role (Benvenuti et al., 2023; Bhatt & Muduli, 2024). A substantial body of research has previously underscored the moderating influences of organizational support, corporate culture, and attachment anxiety on the experience of loneliness (Ernst et al., 2022; Luhmann et al., 2023; Sullivan & Bendell, 2023). However, the present study suggests that, in terms of interpersonal interaction, employees may actively seek additional interpersonal support to alleviate feelings of isolation. In such circumstances, the role of leaders becomes paramount, as they are instrumental in providing emotional resource support, thereby facilitating employees’ restoration of their emotional reserves (Dong & Geng, 2023; Hollebeek et al., 2023). Therefore, the second objective of this research is to assist management in identifying effective response measures to prevent employees from maintaining low levels of emotional resources as a result of AI collaboration. This research corroborates the alleviating effect of leader emotional support on employee loneliness, indicating that human resource managers and organizational leaders must attend to employees’ emotional needs and provide them with emotional support in a timely manner. It is recommended that managers provide emotional care and motivation for employees, pay attention to their social needs, and take measures to alleviate workplace loneliness. Such as providing emotional support, increasing team-building activities, or providing dedicated social spaces to help employees manage their emotional resources more effectively, reduce loneliness generated by AI collaboration, avoid emotional fatigue, and prevent potential negative impacts on subsequent work performance and behavior.

### 4.1. Theoretical Implications

First, this research combines COR theory with employee–AI collaboration to validate the applicability of the theory in explaining employee emotional and behavioral changes, thereby extending the COR theory to the field of AI. The findings of the research suggest that the effect of AI collaboration on employee behavior aligns with the fundamental tenets of the COR theory—the process of resource consumption, that is, the scarcity of resources can trigger negative emotions and behavioral reactions among employees (Dong & Geng, 2023; Hollebeek et al., 2023). This provides a new theoretical perspective for exploring the impact of AI collaboration on individual emotions and behaviors in the future. At the same time, this research examined the effect of employee–AI collaboration on CWB, revealing the potential negative effects of AI collaboration on employees’ work behavior and supplementing relevant research on the antecedents of CWB. By confirming the negative impact of employee–AI collaboration on CWB, it helps deepen our understanding of the impact of employee–AI collaboration and its psychological mechanisms on CWB. Therefore, this study not only expands the research on COR theory and CWB, but also provides a theoretical basis for organizations to prevent negative employee behavior when introducing AI collaboration.

Second, this research examined the effect pathways of employee–AI collaboration on CWB from an emotional perspective, thereby expanding the application scope of COR. AI is inherently incapable of replicating the essential features of human social interaction, thereby impeding the provision of interaction and emotional feedback to employees (Dietvorst et al., 2018; Tang et al., 2023). Employees may perceive that their interactions with AI colleagues significantly exceed their communication and interaction with human colleagues during AI collaboration (Tang et al., 2023). Previous research has explored the effect of AI on employee emotions, drawing upon affective events theory, attachment theory, and related frameworks (Duan et al., 2024; Tang et al., 2023). This research posits that the phenomenon of loneliness stemming from AI can be attributed to diminished emotional resources and an incapacity to access supplementary resources for employees. Furthermore, an investigation was conducted into the exacerbation of employee emotional fatigue caused by loneliness, which further confirms the resource consumption path in COR theory. Those experiencing loneliness often report elevated levels of psychological pressure and emotional loss. Sustained negative emotional state has been shown to erode psychological resources, leading to emotional fatigue (Halbesleben et al., 2014; Hobfoll, 1989). Therefore, according to the COR theory, this research reveals the chain-mediated pathway of loneliness and emotional fatigue and contributes to the antecedents of CWB. At the same time, the research underscores the effect of AI collaboration on employees’ social needs, thereby emphasizing the imperative for preserving interpersonal interaction in the workplace. This theoretical framework provides theoretical guidance for necessary socialization in the workplace.

Third, this research validated the mitigating effect of leader emotional support on the relationship between employee–AI collaboration and loneliness, further enriching the theoretical boundaries of COR theory. In the contemporary workplace, where AI is becoming increasingly prevalent and employee collaboration with AI is becoming the norm, the issue of loneliness experienced by employees due to technological changes is becoming increasingly salient (Tang et al., 2023). This means that employees are unable to obtain emotional resources from others, and their own negative emotions will further deepen their resource depletion. According to the COR theory, when employees’ resources are at a low level, they may seek alternative methods to supplement resources (Dong & Geng, 2023; Hollebeek et al., 2023). In the context of interpersonal interaction within the workplace, employees may proactively seek assistance from their leaders as a means of mitigating feelings of isolation. Concurrently, the emotional resource support provided by leaders functions as a direct conduit to assist employees in replenishing their emotional reserves. Leader emotional support provides psychological comfort, care, and motivation to employees, enhances their sense of belonging and security, thereby reducing loneliness (Halbesleben, 2006; Ito & Brotheridge, 2003; Kock et al., 2018; M. Stein et al., 2021). Consequently, this study offers a theoretical framework for managers to comprehend the role of social interaction and emotional communication by substantiating the regulatory function of leadership emotional support.

### 4.2. Practical Implications

First, organizations need to possess keen insight into the potential CWB that may arise during the process of employee and AI writing. They must implement corresponding measures to reduce the CWB. For employees, AI collaboration signifies a novel modality of work (Sowa et al., 2021; Vrontis et al., 2022). Organizations should, therefore, devise employee training programs to enhance their knowledge and experience related to it, and improve their operational skills in AI collaboration. For instance, organizations can implement targeted training programs to equip employees with the necessary skills to collaborate with AI effectively and to understand its role and application in collaboration (Kong et al., 2023). It establishes a foundation for employees to identify and adapt to their work roles. Organizations should establish comprehensive channels for employee feedback. Employees should be encouraged to share their challenges and difficulties encountered while collaborating with AI, thereby enabling the organization to adjust its strategies timely manner and optimize the mode of employee–AI collaboration. This will motivate employees to proactively engage in AI collaboration, thereby ensuring the organization’s sustained progress in the realm of AI. This will facilitate the attainment of a mutually beneficial development for both employees and the organization.

Second, this research calls on companies to proactively implement measures to assist employees in coping with the emotional effects of AI collaboration on their mental health and behavioral responses (Ashkanasy & Daus, 2002). Organizations must recognize the potential effect of AI collaboration on employee social interaction and emotional states (Wu et al., 2024). This is to avoid employees engaging in harmful behaviors towards the organization due to poor emotional states, such as CWB. In particular, organizations can implement a comprehensive and professional mental health support system, comprising measures such as the establishment of counseling hotlines, the provision of online resources, and the organization of mental health lectures and seminars, in order to address the diverse emotional needs of employees. Organizations may consider the creation of diverse social activity platforms, such as virtual social spaces, interest communities, and team-building activities. This enables employees to obtain timely emotional resource supplementation, thus preventing feelings of loneliness and sustained low-level resource states. Furthermore, organizations can optimize the human–AI collaboration interface and processes. By integrating advanced technologies such as natural language processing and emotion recognition, AI can be rendered more humane and intelligent, thereby facilitating a deeper comprehension of employees’ intentions and emotions, and enabling the delivery of more intimate and personalized services and support (Huang & Rust, 2023; J. Zhang et al., 2024).

Finally, it is incumbent upon managers to provide employees with the emotional care and motivation they require. This can be achieved by fostering regular communication, whether in the form of one-on-one interactions, team meetings, or other avenues, with the aim of gaining insight into the psychological state and emotional needs of the workforce. At the same time, managers can facilitate effective communication with employees through the implementation of regular communication channels. This is not merely about comprehending the status of work and projects; rather, it is of greater importance to listen to the voices of employees and to be mindful of their individual emotional states. By utilizing various forms of communication, including in-person, video conferencing, and instant messaging, managers can promptly identify and address the confusion, challenges, and emotional fluctuations that employees may experience in AI collaboration. At this juncture, it is imperative for managers to demonstrate concern and understanding for employees’ personal emotional issues (Wu et al., 2024). This can be achieved by sharing personal experiences, providing emotional regulation skills, or directly offering encouragement and praise to assist employees in adjusting their mentality and restoring the emotional resources and states necessary for optimal work performance.

### 4.3. Limitations and Future Directions

First, this research did not limit the types of AI in the workplace. Given the heterogeneity of tasks, employees may need to collaborate with different types of AI when dealing with different tasks. In the service industry, for instance, frontline employees collaborate with AI robots to deliver services to customers (Hai et al., 2025; Wu et al., 2025). In innovative work, creative professionals draw inspiration from generative AI. The combination of human customer service and conversational AI to address user inquiries has also emerged (Marvi et al., 2025; Yin et al., 2024). The characteristics of AI vary across different task types, potentially influencing employees’ perceptions and experiences. It is recommended that future research differentiate between different forms of AI and verify the perception and behavioral changes of employees in collaboration with AI by selecting specific occupational types or jobs.

Second, this research only focuses on the effect of employee–AI collaboration on loneliness, without exploring other resources that may be affected by AI collaboration. As an emerging technology, the penetration of AI into employees’ work and life extends far beyond the single dimension of loneliness. We strongly urge and encourage future researchers to delve deeper into the effect of employee–AI collaboration on other resources. For instance, subsequent research endeavors may concentrate on the alterations in employees’ emotional states during collaboration with AI, encompassing the stimulation of positive emotions and the alleviation of negative emotions (Duan et al., 2024; Wu et al., 2022). Additionally, it is feasible to investigate the modifications in employees’ work attitudes, such as whether their level of engagement, sense of responsibility, and professional identity have undergone changes due to the intervention of AI (Cao et al., 2023; Chowdhury et al., 2022; G. Zhang et al., 2023). Furthermore, research can be conducted on the allocation and utilization of work resources, including whether AI has altered the methods and efficiency with which employees access information, technical support, and career development opportunities (Bankins et al., 2024; Huo et al., 2025). By examining these aspects, future research endeavors will be better equipped to elucidate the intricate impact mechanisms of employee–AI collaboration, thereby offering support for organizations to address the challenges posed by the AI collaboration.

Third, this research primarily considered the moderating effect of leader emotional support, defined as assistance from others, without incorporating moderating variables pertaining to the employees themselves. It is recommended that subsequent research explore the impact of a more extensive range of moderating variables. For instance, the examination of how informal learning, social support, organizational training, and intervention measures positively influence employees’ adaptation to AI collaboration could be a particularly relevant area for further inquiry (Benvenuti et al., 2023; Bhatt & Muduli, 2024). In addition to the consideration of external resource support, future research can incorporate other individual-level internal factors, such as openness, emotional attachment, and sense of belonging, to examine the moderating effect of internal resources on the relationship between AI collaboration and employee work experience (Barford & Smillie, 2016; Cugno et al., 2021; Q. Zhang et al., 2025).

## 5. Conclusions

The results of our research indicate that the employee–AI collaboration in the workplace can, inadvertently, lead to an increase in feelings of loneliness among employees, which in turn exacerbates their emotional fatigue. In particular, the interaction between employees and AI has the potential to accentuate feelings of isolation and loneliness among employees, as they may perceive a reduction in the sense of human connection and camaraderie within their work environment. This heightened sense of loneliness serves as a catalyst for emotional fatigue. This emotional toll, when left unchecked, has been demonstrated to be intricately linked to an increased likelihood of engaging in CWB. Such behaviors may range in severity from minor acts of sabotage to more severe forms of misconduct, and can have a significant detrimental impact on organizational culture and performance. However, the research also identifies a crucial positive influence—leader emotional support. It was found that when leaders proactively provide emotional support to employees, it serves as an effective protective measure against the adverse emotional effects associated with AI collaboration. The provision of leader emotional support, which encompasses empathy, understanding, and encouragement, effectively mitigates the loneliness experienced by employees, thereby reducing their emotional fatigue.

## Figures and Tables

**Table 1:** Descriptive statistics and correlations.

Variables	M	SD	1	2	3	4	5	6	7
1 Gender	1.707	0.457	1						
2 Age	29.976	6.730	0.074	1					
3 Education	4.174	0.711	0.213 **	0.157 *	1				
4 Tenure (of years)	6.249	5.631	0.015	0.930 **	0.043	1			
5 Loneliness	2.689	1.205	−0.069	−0.032	−0.084	0.005	1		
6 Emotional fatigue	3.999	1.687	−0.077	−0.128	−0.171 *	−0.057	0.835 **	1	
7 Counterproductive work behavior	2.465	1.241	0.026	−0.167 *	−0.044	−0.132	0.384 **	0.463 **	1

Note: N = 167, * *p* < 0.05, ** *p* < 0.01.

**Table 2:** Regression analysis of mediator model.

Variable	Loneliness				Emotional Fatigue				CWB			
	B	SE	B	SE	B	SE	B	SE	B	SE	B	SE
Control												
Gender	−0.045	0.212	−0.034	0.210	−0.018	0.289	0.011	0.160	0.076	0.199	0.071	0.193
Age	−0.207	0.040	−0.207	0.040	−0.444 *	0.055	−0.274 *	0.030	−0.265	0.038	−0.142	0.037
Education	−0.051	0.142	−0.043	0.140	−0.105	0.193	−0.070	0.107	0.015	0.133	0.046	0.130
Tenure (of years)	0.200	0.047	0.206	0.047	0.364	0.065	0.195	0.036	0.116	0.045	0.028	0.044
Independent variable												
E-AIC			0.157 *	0.186	0.121	0.257	−0.009	0.143	0.133	0.179	0.136	0.173
Mediator												
Loneliness							0.822 *	0.060	0.359 ***	0.075	−0.011	0.131
Emotional fatigue											0.450 ***	0.095
*R* ^2^	0.015		0.039		0.071		0.720		0.194		0.250	
Δ*R*^2^	−		0.024 *		−		0.649 *		−		0.056 **	
*F*	0.617		1.323		2.478*		68.536 ***		6.399 *		7.584 ***	

Note: N = 167, * *p* < 0.05, ** *p* < 0.01, *** *p* < 0.001. E-AIC = employee–AI collaboration; CWB = counterproductive work behavior.

**Table 3:** Path analysis of mediator model.

	Effect	BootSE	BootLLCI	BootULCI
Indirect effect				
E-AIC → Loneliness → CWB	−0.003	0.051	−0.103	0.108
E-AIC → Emotional fatigue → CWB	−0.008	0.040	−0.093	0.069
E-AIC → Loneliness → Emotional fatigue → CWB	0.116	0.066	0.004	0.257
Total effects	0.105	0.075	−0.035	0.260

Note: N = 167. E-AIC = employee–AI collaboration; CWB = counterproductive work behavior.

## Data Availability

Data and Materials for this study are available by emailing the corresponding author. The code behind this analysis/simulation is not available because it is proprietary.
